# CD46 and CD59 inhibitors enhance complement-dependent cytotoxicity of anti-CD38 monoclonal antibodies daratumumab and isatuximab in multiple myeloma and other B-cell malignancy cells

**DOI:** 10.1080/15384047.2024.2314322

**Published:** 2024-02-15

**Authors:** Hongjie Wang, Theo Koob, Jonathan R. Fromm, Ajay Gopal, Darrick Carter, André Lieber

**Affiliations:** a Department of Medicine, University of Washington, Seattle, WA, USA; b Department of Laboratory Medicine and Pathology, University of Washington, Seattle, WA, USA; c R&D, Compliment Corp, Seattle, WA, USA

**Keywords:** Multiple myeloma, daratumumab, isatuximab, complement dependent cytotoxicity, resistance

## Abstract

Multiple myeloma (MM) is an incurable malignancy of the B-cell lineage. Remarkable progress has been made in the treatment of MM with anti-CD38 monoclonal antibodies such as daratumumab and isatuximab, which can kill MM cells by inducing complement-dependent cytotoxicity (CDC). We showed that the CDC efficacy of daratumumab and isatuximab is limited by membrane complement inhibitors, including CD46 and CD59, which are upregulated in MM cells. We recently developed a small recombinant protein, Ad35K++, which is capable of transiently removing CD46 from the cell surface. We also produced a peptide inhibitor of CD59 (rILYd4). In this study, we tested Ad35K++ and rILYd4 in combination with daratumumab and isatuximab in MM cells as well as in cells from two other B-cell malignancies. We showed that Ad35K++ and rILYd4 increased CDC triggered by daratumumab and isatuximab. The combination of both inhibitors had an additive effect *in vitro* in primary MM cells as well as *in vivo* in a mouse xenograft model of MM. Daratumumab and isatuximab treatment of MM lines (without Ad35K++ or rILYd4) resulted in the upregulation of CD46/CD59 and/or survival of CD46^high^/CD59^high^ MM cells that escaped the second round of daratumumab and isatuximab treatment. The escape in the second treatment cycle was prevented by the pretreatment of cells with Ad35K++. Overall, our data demonstrate that Ad35K++ and rILYd4 are efficient co-therapeutics of daratumumab and isatuximab, specifically in multi-cycle treatment regimens, and could be used to improve treatment of multiple myeloma.

## Introduction

Multiple myeloma (MM) is characterized by the neoplastic monoclonal expansion of plasma cells in the bone marrow. Progress has been made in the treatment of MM with the introduction of immunomodulatory drugs, proteasome inhibitors, and recently with monoclonal antibodies and CAR T cells (for review.^1^ Therapeutic monoclonal antibodies (mAbs) include daratumumab (Darzalex) and isatuximab (Sarclisa). These mAbs bind to different epitopes of CD38,^2^ a receptor that is uniformly overexpressed on MM, but the degree of overexpression of CD38 varies in MM patients.^3–5^ Both mAbs can cause cell death by complement-dependent cytotoxicity (CDC),^6,7^ among other mechanisms.^8^ Daratumumab is usually administered in combination with bortezomib/lenalidomide and dexamethasone^9,10^ in transplant-ineligible patients with progressive disease and, more recently, also in frontline settings. Isatuximab is often administered after unsuccessful daratumumab treatment.^11^ Most patients develop progressive disease after anti-CD38 mAb therapy, and their prognosis is poor.^12^ For example, in a multicenter study that examined 107 patients with relapsed/refractory MM on daratumumab monotherapy, the overall response rate was 42.1%, and the median first and second progression-free survival were 3.6 and 8.1 months, respectively, with an overall survival of 11.9 months in this group.^13^ The overall response rate was 46.2% in the patients who received isatuximab after daratumumab treatment. The 1-year overall survival rate was 53.9%, with a median progression-free survival of 5.6 months.^11^

CD38 is also expressed on normal white blood cells, although at lower levels than on MM cells.^1^ Daratumumab and isatuximab can trigger neutropenia or thrombocytopenia, which often requires mAb dose reduction or a halt of the treatment.

Binding of C1q to the Fc tail of the therapeutic mAb initiates the complement cascade, ultimately resulting in the generation of a membrane attack complex (MAC) and subsequent permeabilization of the cell membrane.^14^ The deposition of complement components such as C3b on the surface of the target cell is also a consequence of complement activation. These deposited complement components interact with complement receptors on phagocytic cells, resulting in engulfment of tumor cells. A series of complement regulatory proteins, including CD46 and CD59, protects healthy tissues against accidental complement attacks. These complement inhibitors have also been shown to protect tumor cells against several therapeutic antibodies.^15^ CD46 has cofactor activity for inactivation of complement components C3b and C4b by serum factor I,^16^ whereas CD59 binds to C8 and C9, preventing C9 polymerization and insertion into membranes.^17^ There is substantial evidence in the literature that tumors^18–22^ - including MM^23–25^ - overexpress CD46 and CD59 and that these proteins limit the efficacy of mAb therapy.^26^

Here, we employed two small recombinant proteins, Ad35K++ and rILYd4, to eliminate or block CD46 and CD59, respectively, with the goal of increasing the efficacy of CDC triggered by daratumumab and isatuximab in MM cells.

Ad35K++ is a small, recombinant protein that is cost-efficiently produced in bacteria. It is derived from an adenovirus serotype 35.^27^ Ad35 engages CD46 via residues in its C-terminal trimeric fiber knob domain. A series of mutations was introduced into the recombinant fiber knob protein to enhance the affinity of the trimer. The recombinant fiber knob protein with the highest affinity (*K*_D_ = 0.63 nM) was named Ad35K++.^27^ Ad35K++ crosslinks several CD46 proteins on the membrane of cancer cells, leading to shedding of the extracellular domain of CD46 and its internalization.^28^ We demonstrated that Ad35K++ increased the efficacy of lymphoma cell killing by the anti-CD20 mAb rituximab *in vitro* both in primary and established human CD20-positive lymphoma/leukemia cells and *in vivo* in tumor xenograft models.^28^ Studies in non-human primates showed that the combination of rituximab and Ad35K++ was able to eliminate B cells, whereas treatment with the same dose of rituximab alone had no effect.^29,30^ These primate studies have also demonstrated that the therapy is safe and well-tolerated.^29^ Ad35K++ has been produced as a cGMP grade product, and the combination of Ad35K++ and rituximab for the treatment of B-cell malignancies has an open IND with the US FDA and awaits clinical testing in humans.

rILYd4 is a CD59 inhibitor that was developed by Dr. Xuebin Qi’s group at the Harvard Medical School.^31^ rILYd4 consists of a 114-amino acid recombinant form of the 4th domain (d4) of intermedilysin, a pore-forming toxin secreted by *Streptococcus intermedius*. rILYd4 binds to CD59 but does not induce cell lysis.^31^ Upon rILYd4 binding, CD59 is internalized and undergoes degradation in the lysosomes within minutes. The remaining rILYd4·CD59 complexes recycle to the cell membrane and are shed from the cell.^32^ rILYd4 enhanced CDC triggered by the anti-CD20 mAbs ofatumumab or rituximab in rituximab-resistant lymphoma cells and primary chronic lymphocytic leukemia cells *in vitro*.^33^

## Results

### CD38, CD46, and CD59 expression on test cell lines

**Figure 1.**
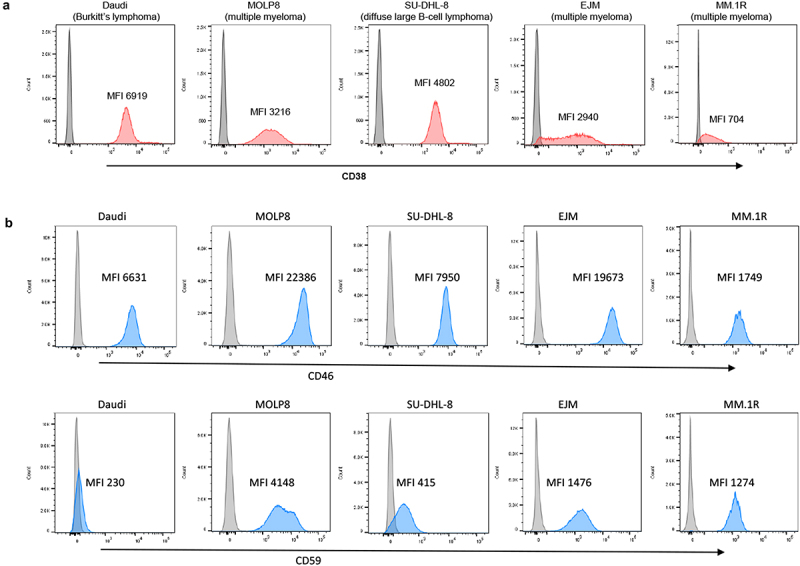
Flow cytometry analyses for CD38, CD46 and CD59. Daudi cells, SU-DHL-8, MOLP8, EJM, and MM.1 R cells were incubated with fluorophore-labeled anti-CD38, CD46 or CD59 antibodies. a) CD38. The gray peak in the flow histogram is the negative control (without antibody staining). The pink area represents cells stained with anti-CD38 antibody. b) CD46 and CD59. The gray curve is the negative control (without antibody staining). The blue curve represents cells detected by anti-CD46 or anti-CD59 antibodies. Shown is also the mean fluorescence intensity (MFI) of CD38, CD46 and CD59.

Our studies were performed using the MM cell lines MOLP8, EJM, and MM.1 R. Because daratumumab and isatuximab can also target other CD38-positive B-cell malignancies, we included Daudi cells (Burkitt’s lymphoma) and SU-DHL-8 (diffuse large B cell lymphoma) in our study. CD38 expression, analyzed using flow cytometry, was uniformly high in Daudi, MOLP8, and SU-DHL-8 cells (Figure 1a). CD38 expression levels in EJM cells varied, with ~ 5% of the cells being CD38-negative. A similar pattern was seen in MM.1 R cells, which also had a lower CD38 mean fluorescence intensity (MFI) than EJM cells. In addition to CD38 levels, the levels of the complement inhibitors CD46 and CD59 were measured. Flow cytometry showed uniformly high expression of CD46 on all test cell lines with the highest MFIs (*i.e*., expression levels) in MOLP8 cells followed by EJM, SU-DHL-8, Daudi and MM.1 R (Figure 1b, upper panel). CD59 expression was absent on Daudi cells implying that rILYd4 will probably not have an effect on these cells. In the other test cell lines, the levels of CD59 were the highest in MOLP8 cells, followed by EJM, MM.1 R, and SU-DHL-8.

### The CD46 inhibitor Ad35K++ increases CDC efficacy of daratumumab and isatuximab

A cGMP grade Ad35K++ protein preparation was used in these studies.^30^ Size exclusion chromatography demonstrated the Ad35K++ homotrimer has > 95% purity with less than 5% aggregate (Fig.S1A). The reduced 4–15% gradient SDS-PAGE gel shows the homotrimer that migrates around 68 kDa when the sample is not boiled, due to the remarkable energetic favorability of the trimeric state (Fig.S1B). The trimeric state is only disrupted on boiling in SDS sample buffer, after which it migrates at an apparent molecular weight of ~ 23 kDa. The identity of the protein bands is confirmed by Western blot analysis (Fig.S1C), where the blotted Ad35K++ is incubated with soluble CD46 and then detected with an anti-CD46 antibody.

rILYd4 was produced in *E. coli* and purified using an N-terminal His-tag. SDS polyacrylamide electrophoresis showed that the recombinant protein had the correct molecular weight and purity of > 90% (Fig.S2A). The CDC-enhancing activity of rILYd4 was validated in a CDC assay with rituximab, an anti-CD20 mAb used in our previous studies^28–30^ (Fig.S2B).

**Figure 2.**
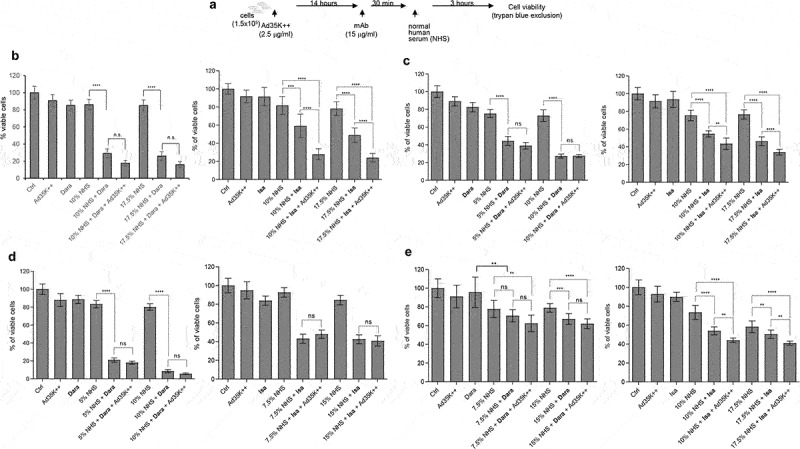
Effect of Ad35K++ preincubation on complement-dependent-cytotoxicity (CDC) triggered by daratumumab or isatuximab. a) schematic of the experiment. 1.5 × 10^5^ cells/sample were preincubated with PBS or 2.5 μg/mL Ad35K++ knob protein for 14 hours. Daratumumab (Dara) or isatuximab (isa) (15 μg/ml) were added to cells and incubated at room temperature for 30 minutes. Then, normal human serum (NHS) was added, and cells were incubated at 37°C for another 3 hours. Viable cells in each well were counted after trypan blue staining. b) studies with Daudi cells. The left graph shows data with daratumumab. The right graph shows results with isatuximab. Two different NHS concentrations were tested. c) studies with MOLP8 cells. d) studies with SU-DHL-8 cells. e) studies with EJM cells. For each data set, three independent experiments with three technical replicas were performed. ** *p* < .05, ***p < .01, **** *p* < .001, n.S. not significant.

Ad35K++ was tested in combination with daratumumab and isatuximab (Figure 2). The test cells were preincubated with or with 2.5 μg/ml Ad35K++ at a concentration that was found to be effective in preliminary titration studies (Fig.S3). Fourteen hours later, daratumumab or isatuximab were added at a concentration of 15 μg/ml (Figure 2a). We used the same mAb concentration as that used in our previous studies with rituximab. Thirty minutes after incubation with the mAbs, normal human serum (NHS) was added as the source of complement factors. (NHS is pooled from 400–500 healthy donors). Three hours after incubation with NHS, viable cells were counted using trypan blue exclusion.

Studies with Daudi cells are shown in Figure 2b. Incubation with daratumumab or isatuximab at 15 μg/ml did not trigger significant cell death. This required the presence of NHS. Daratumumab triggered efficient CDC that could not be significantly increased by preincubation with Ad35K++. Isatuximab at 15 μg/ml triggered less CDC than daratumumab, and, importantly, Ad35K++ significantly increased isatuximab cell killing. In MOLP8 cells, similar to Daudi cells, daratumumab triggered efficient CDC that was not enhanced by Ad35K++, while Ad35K++ significantly increased isatuximab CDC (Figure 2c). In SU-DHL-8 cells, no significant enhancing effect of Ad35K++ was observed for either mAb when used at a concentration of 15 μg/ml (Figure 2d). In EJM cells, daratumumab- and isatuximab-triggered CDC was less than that in the other cell lines, most likely due to lower CD38 expression (Figure 2e). Ad35K++ significantly increased isatuxumab-induced CDC in EJM cells.

**Figure 3.**
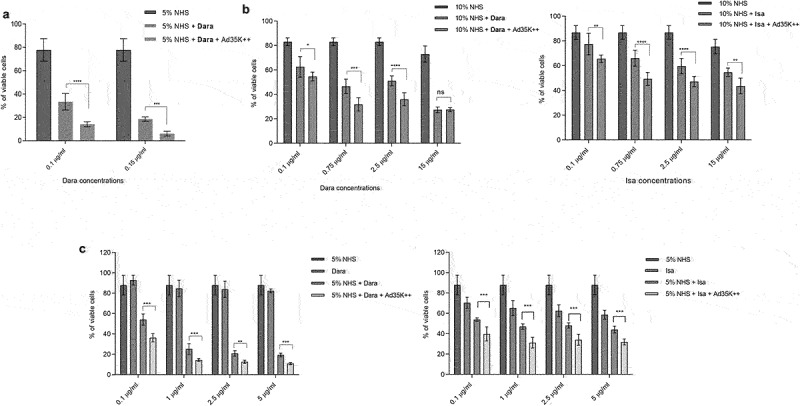
Ad35K++ preincubation increased CDC when test cells are incubated with daratumumab or isatuximab at concentrations that were lower than 15 μg/ml. The studies were performed as described in (Fig. 2a). a) Daudi cells. Shown are the percentages of viable cells in settings that were incubated with 10% NHS, NHS+daratumumab, and NHS+daratumumab+Ad35K++. The daratumumab concentrations are shown below the x-axis. The enhancing effect of Ad35K++ on daratumumab-triggered CDC (“Nhs+dara” “NHS+Dara+Ad35K++”) was significant for all both concentrations (*** *p* < .01). b) studies in MOLP8 cells with lower concentrations of daratumumab or isatuximab. The CDC-enhancing effect of Ad35K++ was significant (*** *p* < .01). c) studies in SU-DHL-8 cells with lower concentrations of daratumumab or isatuximab. The CDC-enhancing effect of Ad35K++ was significant (*** *p* < .01). For each data set, three independent experiments with three technical replicas were performed.

Overall, in most of these settings, a concentration of 15 μg/ml daratumumab appeared to saturate CDC induction. Therefore, we performed CDC assays using lower concentrations of daratumumab and isatuximab. These studies are clinically relevant considering that during multi-cycle treatment of patients with MM, daratumumab doses often have to be lowered due to side effects. In Daudi cells that were treated with daratumumab at concentrations of 0.1 and 0.15 μg/ml, Ad35K++ preincubation significantly increased CDC (Figure 3a, “NHS + Dara” vs “NHS + Dara + Ad35K++”). In MOLP8 cells, Ad35K++ significantly enhanced CDC when cells were incubated with lower daratumumab concentrations (2.5, 0.75, and 0.1 μg/ml) (Figure 3b, left panel). A similar tendency was observed for the combination of Ad35K++ and isatuximab (Figure 3b, right panel). Ad35K++ also enhanced CDC at lower concentrations of daratumumab and isatuximab (5, 2.5, 1, 0.1 μg/ml) in SU-DHL-8 cells (Figure 3d).

These data indicate that Ad35K++ could salvage the therapeutic effect of daratumumab and isatuximab when dose reduction is required or may allow for longer dosing intervals while maintaining efficacy.

### Ad35K++ and CD59 inhibitor (rILYD4) additively enhance CDC by daratumumab and isatuximab in MM cells

**Figure 4.**
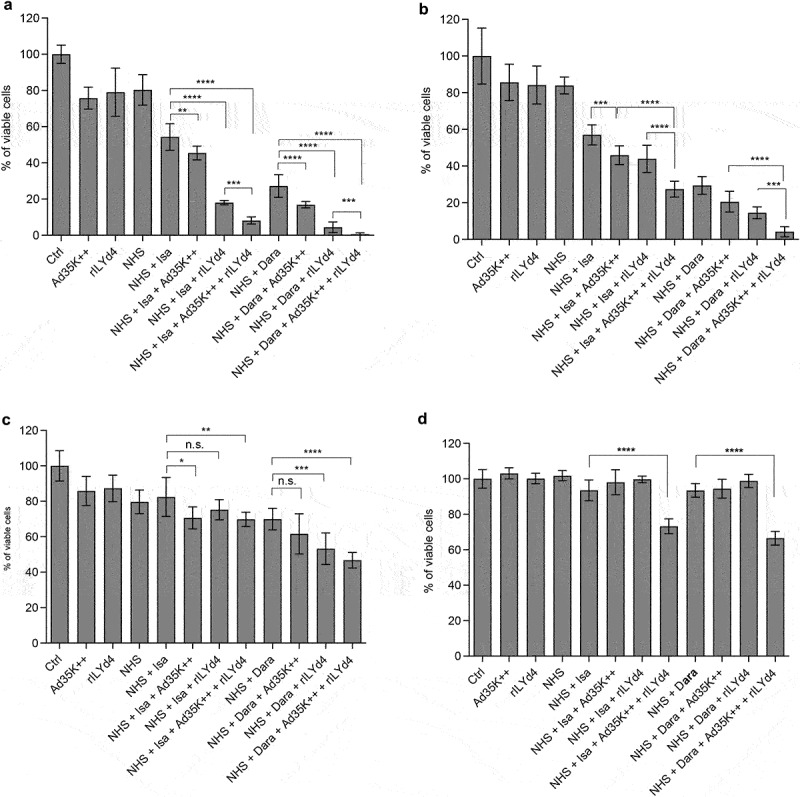
The combination of Ad35K++ and rIYd4 additively enhanced the CDC effect of daratumumab and isatuximab. MOLP8 (a), SU-DHL-8 (b), EJM (c) and MM.1 R (d) cells were preincubated with PBS, or 2.5 μg/mL Ad35K++ and/or rILYd4 protein. 14 hours later, daratumumab (Dara, MOLP8, SU-DHL-8, and MM.1 R: 2.5 μg/ml, EJM: 15 μg/ml) or isatuximab (MOLP8, SU-DHL-8, and MM.1 R: 2.5 μg/ml, EJM: 15 μg/ml) were added to cells and incubated at room temperature for 30 minutes. 10% NHS was then added, and cells were incubated at 37°C for another 3 hours. Viable cells in each well were counted after trypan blue staining. For each data set, three independent experiments with three technical replicas were performed. ** *p* < .05, ***p < .01, **** *p* < .001, n.S. not significant.

We hypothesized that simultaneous inhibition of two of the major complement regulatory proteins, CD46 and CD59, would have a synergistic effect on CDC triggered by daratumumab and isatuximab in MM cells. Therefore, we tested the CDC-enhancing effect of preincubation with Ad35K++ and the CD59-inhibitor rILYd4 alone and in combination (Figure 4). In MOLP8 cells, the MM cell line with the highest level of CD59 expression, preincubation with rILYd4 exerted a significantly stronger enhancement of daratumumab- and isatuximab-triggered CDC than preincubation with Ad35K++ (Figure 4a). Importantly, preincubation with both inhibitor proteins had an additive effect, resulting in 92% and 99.4% CDC killing of MOLP8 cells with isatuximab and daratumumab, respectively. In SU-DHL-8 cells, both Ad35K++ and rILYd4 increased daratumumab- and isatuximab-mediated CDC to a similar level (Fihg.4B). Again, the combination of both had an additive effect, with 72.6% and 96% cells killed by isatuximab and daratumumab, respectively. An additive effect of Ad35K++ and rILYd4 was also demonstrated in EJM cells, although in this cell line, a maximum of 54% of cells could be killed by anti-CD38 mAbs (Figure 4c). Studies in MM.1 R cells, the MM cell line with the lowest C38 MFI showed daratumumab- and isatuximab-triggered CDC only if cells were pre-incubated with the combination of Ad35K++ and rILYD4 (Figure 4d).

**Figure 5.**
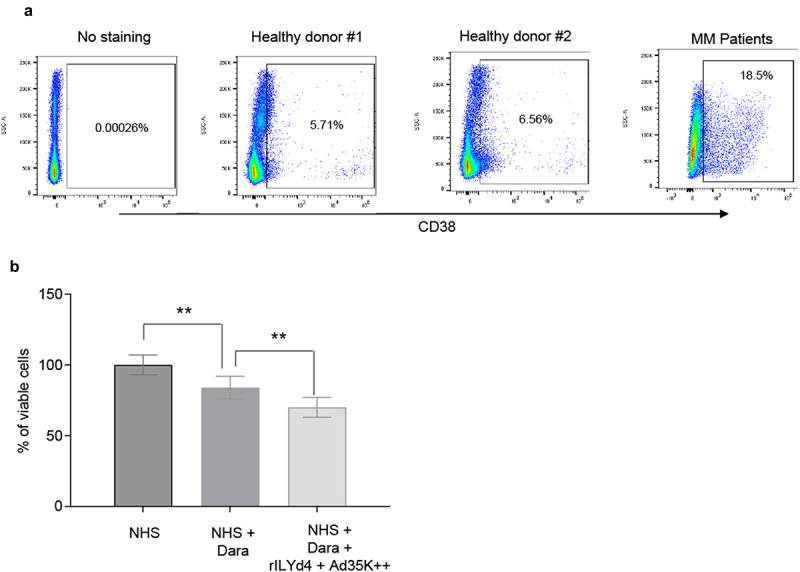
CDC in patient MM cells triggered by daratumumab. a) PBMCs from two healthy donors and from five MM patients were analyzed by flow cytometry for CD38 expression. Cells from MM patients were pooled because of low cell numbers. b) CDC assay with pooled patient MM cells. MACS-isolated CD38+ primary MM cells were incubated 2.5 µg/ml Ad35K++ and rILYd4 protein. 14 hours later, daratumumab (Dara, 2.5 µg/ml) was added to cells and incubated at room temperature for 30 minutes. 10% normal human serum (NHS) was then added, and cells were incubated at 37°C for another 3 hours. Viable cells in each well were counted after trypan blue staining. Three technical replicates were analyzed. *p* values: ** p < .05.

For CDC studies in primary MM cells, peripheral blood mononuclear cells of five MM patients were used. The percentage of CD38^+^ cells in these samples varied from 8.1 to 38%. The percentage of CD38^+^ cells from two healthy donors was on average 6.1% (Figure 5a). Because of low cell numbers, patient samples were pooled. The pool contained 18.5% CD38^+^ cells (Figure 5a). Magnetic Activated Cell Sorting was used to isolate CD38^+^ cells (purity >90%). These cells were then subjected to CDC assay with daratumumab. Daramatumab triggered significant CDC, which was further significantly increased by pre-incubation with Ad35K++ and rILYd4 (Figure 5b).

### Daratumumab and isatuximab treatment of MM cell lines results in upregulation of CD46 and CD59 and escape during a second mAb treatment round. Escape can be reduced by pretreatment with Ad35K++ and/or rIlyd4

**Figure 6.**
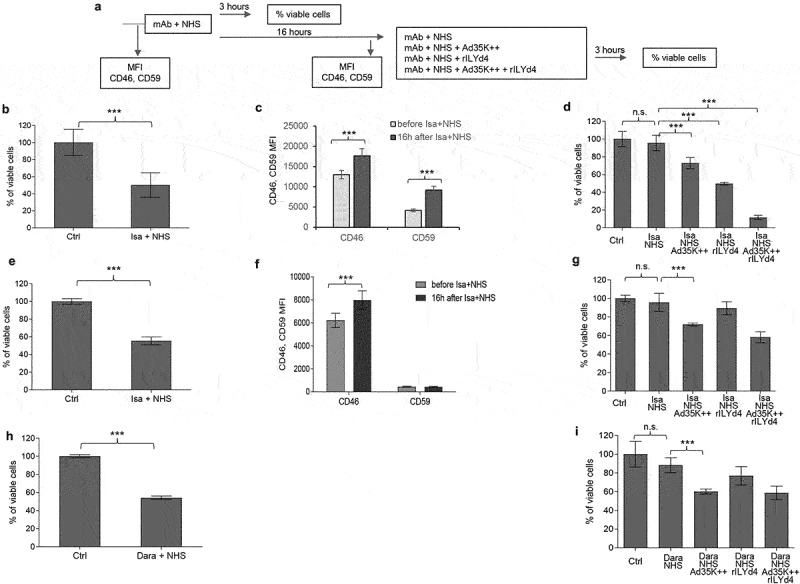
Co-treatment of MOLP8 cells with Ad35K++ and rILYd4overcomes resistance to isatuximab after the second cycle of treatment in MOLP8 and SU-DHL-8 cells. a) schematic of the experiment. Test cells (MOLP8 and SU-DHL8) were incubated with isatuximab/daratumumab in the presence of NHS to trigger CDC. CD46 and CD59 MFIs were measured before and 16 hours after adding mAb/NHS (c and f). Cell viability was counted 16 hours after mAb/NHS (b, e, h). Cells were then either incubated with or without Ad35K++/rILYd4 and isatuximab or daratumumab (d, g, i). (b-d) studies with MOLP8 cells. e-i) studies with SU-DHL-8 cells. For each data set, three independent experiments with three technical replicas were performed. ***p < .01, n.S. not significant.

Daratumumab and isatuximab treatment of MM patients involves multiple treatment cycles; for example, six infusions once weekly during the first six weeks followed by up to 16 cycles every three weeks until week 54, and then weekly infusion until disease progression. Therefore, we developed a study plan that included two rounds of mAb treatment of MM cells (Figure 6a). The test cells were MOLP8 (Figures. 6b–f) and SU-DHL-8. The first treatment with isatuximab/daratumumab + NHS measures the CDC triggered by these antibodies. In agreement with previous studies, both antibodies triggered significant CDC in both cell lines (Figures. 6b, e, h). The MFIs for CD46 and CD59 were recorded before and 16 h after the mAb/NHS treatment. In MOLP8 cells, mAb/NHS treatment induced a significant upregulation of both CD46 and CD59 (Figure 6c). In SU-DHL-8 cells, CD46 levels increased with treatment, while the initially low CD59 levels remained unchanged (Figure 6f). Upregulation of complement inhibitors was most likely causatively involved in the development of resistance to CDC after a second round of treatment with isatuximab/NHS (Figures. 6d, g, first two bars) and daratumumab/NHS (Figure 6i). Importantly, resistance could be overcome by pretreatment with Ad35K++ and/or rILYd4. In MOLP8 cells, Ad35K++ and rILYd4 individually increased isatuximab CDC (Figure 6d). This combination has an additive effect. In SU-DHL-8 cells, Ad35K++ conferred significant CDC by isatuximab (Figure 6g) and daratumumab (Figure 6I). The CDC-enhancing effect of rILYd4 was not significant, indicating that CD59 may not play a role in conferring resistance after repeated mAb treatment in SU-DHL-8 cells, which is in agreement with the absence of CD59 upregulation upon mAb/NHS treatment.

Our data indicate that the efficacy of multiple mAb treatment rounds might be affected by the upregulation of complement inhibitors and that this can, in part, be overcome by co-therapy with Ad35K++ and/or rILYd4.

### Ad35K++ and CD59 inhibitor (rILYD4) additively enhance CDC by daratumumab in vivo in a mouse xenograft model of MM

**Figure 7.**
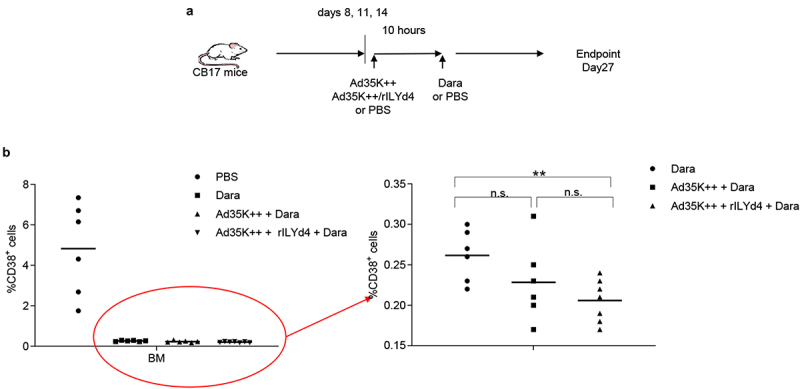
*In vivo* study in a MOLP8 MM xenograft mouse model. a) schematic of the experiment. To establish the xenograft MM model, a total of 2 × 10^6^ MOLP8 cells in 200 μl of PBS were injected into the tail vein of immunodeficient severe combine immunodeficient (Scid)/beige mice. On days 8, 11 and 14, animals were intravenously injected with PBS, 50 µg Ad35K++ and rIlyd4. Daratumumab (Dara) or PBS were given intravenously 10 hours later. The percentage of human CD38^+^ cells in the bone marrow were analyzed on day 27 after injection of MOLP8 cells. b) flow cytometry analysis of human CD38^+^ cells in the bone marrow. Total bone marrow mononuclear cells (BM-MNCs) were isolated on day 27 after injection of daratumumab and analyzed for CD38 expression. The left panel shows the percentage of CD38^+^ cells in BM MNCs in all four groups including the (untreated) control group that received PBS instead of Ad35K++/rILYd4 and instead of daratumumab. Each symbol is an individual mouse. The right panel shows the treatment groups only. Note the new Y-scale. ** *p* < .05.

To establish a MM *in vivo* model, we intravenously injected MOLP8 cells into immunodeficient CB17-SCID/beige mice. Eight days later, we started three rounds of treatment with daratumumab (intravenous) with and without intravenous Ad35++ plus rILYd4 pre-injection (10 hours before Dara) (Figure 7a). Control animals received PBS injections instead of Ad35K++/rILYd4 and daratumumab. On day 27 after MOLP8 injection, when control animals began to show the first signs of sickness (low activity, hunching), all animals were sacrificed. Bone marrow was isolated and bone marrow mononuclear cells (BM-MNCs) were subjected to flow cytometry for human CD38. In the bone marrow of untreated control animals, on average 5% of total BM-MNCs were positive for human CD38. In the groups that received, the percentage of human CD38^+^ cells was less than 0.5% (Figure 7b, left panel). Within the daratumumab treated groups, the percentage of human CD38^+^ cells was significantly lower in mice that received Ad35K++ plus rILYd4 injections prior to daratumumab (Figure 7b, right panel).

## Discussion

Treatment of MM with the CD38 mAbs daratumumab and isatuximab can be effective; however, resistance eventually develops, and the disease progresses. Both antibodies can mediate MM cell death through CDC induction. This was also confirmed in our study, where incubation with mAbs alone had no significant cell killing effect. The presence of complement factors in Normal Human Serum was required to exert significant cell killing. The focus of this study was to enhance CDC triggered by daratumumab and isatuximab in MM cells (MOLP8, EJM, and MM.1 R) and other B-cell malignancies (Daudi and SU-DHL-8) by interfering with the complement inhibitors, CD46 and CD59. In agreement with published studies,^34^ we showed that both proteins are expressed in MM cell lines, whereby the protein level as measured by mean fluorescence intensity varied between cell lines. CD46 levels were higher in MOLP8 cells than in the other cell lines, which could be one of the factors why MOLP8 cells were more resistant to CDC (Figures 3b, c).

Another factor that determines the efficacy of mAb-induced CDC is the level of the target receptor CD38. This became particularly apparent in studies with EJM and MM1.R cells, MM cell lines that display a spectrum of CD38 expression, with most cells being CD38^low/negative^. In these cell lines, daratumumab and isatuximab did not trigger significant CDC without preincubation with Ad35K++ plus rILYd4. This implies that CD38^low^ MM cells can be sensitized to daratumumab by a combination with Ad35K++ and rILYd4. Other studies with clinical samples also found a correlation between the expression levels of CD38 and daratumumab-associated CDC,^34,35^ and approaches to pharmacologically upregulate CD38 levels improve the efficacy of daratumumab.^36^ The density of the surface target also enhances the ability of the complement to kill cells; therefore, this correlation further supports the importance of CDC for daratumumab’s action.

An important finding in our study was that Ad35K++ pretreatment significantly increased the efficacy of daratumumab and isatuximab on MM cells when used at low concentrations (0.1 μg/ml to 2.5 μg/ml). This indicates that Ad35K++ co-treatment could salvage the therapeutic effect, even if mAb dose reduction is required owing to adverse side effects related to the mAb. This feature of Ad35K++ is also supported by our non-human primate studies with rituximab, where Ad35K++ pretreatment converted a subtherapeutic dose of rituximab into a B-cell depleting dose.^30^ Treatment with lower mAb doses or longer intervals between doses is also relevant from a cost perspective.

We found higher CD46 and CD59 levels/MFIs after one round of mAb/NHS treatment of the MM cell lines. This could be the result of the upregulated expression of the corresponding genes. However, considering the existence of MM cell subpopulations with markedly different levels of CD46 and CD59, it is more likely that mAb/NHS treatment was selected for CD46^high^ and CD59^high^ populations, which then contributed to the resistant MM phenotype in the second round of mAb/NHS treatment. Our finding is in line with a report where a marked increase in CD59 levels was observed in both MM cells localized in the bone marrow, as well as in circulating MM cells during daratumumab treatment.^25,37^ Importantly, pretreatment with Ad35K++ during the second cycle of mAb/NHS treatment resulted in significant CDC, indicating that Ad35K++ can partially overcome resistance to mAb treatment. This could be crucial because daratumumab and isatuximab are administered over multiple treatment cycles. In this context, it is noteworthy that CD59 inhibition by CD59 antibodies enabled isatuximab to trigger the killing of MM cells that were otherwise resistant to isatuximab-induced CDC.^26^

We also demonstrated that Ad35K++ plus rILYd4 significantly enhances the therapeutic efficacy of daratumumab in a xenograft MM mouse model. Daratumumab suppressed growth of MOLP8 cells in the bone marrow in *T*- and B-cell deficient mice. This suggests that the therapeutic effect of daratumumab involves CDC and that it can be enhanced by CD46 and CD59 inhibitors.

In our study, we did not compare the effects of anti-CD59 antibodies on CDC triggered by daratumumab and isatuximab in MM cells. Previously, we compared CD46 antibodies against Ad35K++ in combination with the anti-CD20 mAb rituximab.^28^ Preincubation of CD20^+^ Raji cells with anti-CD46 mAb in combination with rituximab/NHS was significantly less efficient in enhancing rituximab CDC than preincubation with Ad35K++. Crimeen-Irwin *et al*. reported that incubation with monoclonal antibodies against CD46 did not trigger long-term CD46 downregulation.^38^ The downstream events of binding anti-CD46 mAb vs. Ad35K++ are different. While mAbs trigger internalization of CD46 through micropinocytosis,^38^ Ad35K++ binding to CD46 initiates cleavage and shedding of the extracellular CD46 domain, a process that involves matrix metalloproteinases.^39^ These differences can be attributed to the specifics of ligand binding. Ad35K++ crosslinks three CD46 receptors with picomolar-binding avidity. Notably, manufacturing Ad35K++ and rYLd4 in *E. coli* is technically easier and cheaper than producing mAbs. This is relevant if CD46 or CD59 inhibition is considered a co-therapy to the already expensive therapy with daratumumab or isatuximab.

In summary, our *in vitro* studies indicate that the efficacy of daratumumab and isatuximab in the treatment of MM was increased by co-treatment with Ad35K++ and/or rILYd4. This approach is also relevant for new anti-CD38 mAbs with enhanced Fc-dependent MM cell depletion.^40^

## Materials and methods

### Cells

Daudi (ATCC-CCL-213) and SU-DHL-8 (CVCL-2961) cells were cultured in RPMI1640 supplemented with 10% heat-inactivated FBS and penicillin/streptomycin. MOLP8 (CVCL-2224) and MM.1 R cells (CRL-2975) cells were cultured in RPMI1640 supplemented with 20% heat-inactivated FBS and penicillin/streptomycin. EJM (CVCL-2030) was cultured in Iscove’s MDM supplemented with 20% heat-inactivated fetal bovine serum (FBS) and penicillin/streptomycin.

Patient peripheral blood mononuclear cells (PMCS) were obtained in the context of University of Washington STUDY00012641 and provided for the studies without patient identification information. After recovery from cryotubes, PBMCs were incubated for 6 hours in RPMI 1640 medium containing 10% FCS, 1 mM sodium pyruvate, 2 mM Hepes, 2.5 µg/ml amphotericin B, 500 µg/ml gentamicin, 2 mM L-glutamine. From the pool of cells, CD38-positive MM cells were isolated by Magnetic Activated Cell Sorting using a CD38 MicroBead Kit (Miltenyi, 130-092-263) and incubated with Ad35K++ and rILYd4 overnight followed by the standard CDC procedure.

### Therapeutic monoclonal antibodies

Daratumumab (Darzalex^R^) (Johnson & Johnson, New Jersey, USA), Isatuximab (Sarclisa^R^) (Sanofi, Paris, France) were purchased from UW Pharmacy. Daratumumab is a human antibody derived from human IgG transgenic mice and isatuximab is a chimeric (mouse/human) antibody.

### Normal human serum (NHS)

NHS was purchased from Sigma-Aldrich (S1, cat. number 637,810, batch number 8567). A typical batch is pooled from 400–500 healthy donors.

### Ad35K++

Ad35K++ was produced in *E coli* with N-terminal tags of six consecutive histidine residues and purified by Ni-NTA agarose chromatography, as described elsewhere.^30^ Fiber knob protein was dialyzed against 20 mM HEPES, 200 mM NaCl, and 17% glycerol. Endotoxin tests were performed using the Limulus Amebocyte Lysate test kit from Cape Cod, Inc. For *in vivo* studies, preparations of recombinant Ad35K^++^ with less than 0.25 EU/mL endotoxin were used.

### Size exclusion chromatography

Samples were loaded onto a Superdex 200 Increase 10/300 GL column (Cytiva Life Sciences) and flowed through an Agilent 1260 series HPLC using 1×Tris-buffered saline (TBS) at a flow rate of 1 mL/min to obtain the elution profiles.

### rILYd4 production

The rILYd4 fragment (GenBank accession number AB029317) was synthesized using Genscript (Nanjing, China) and cloned into pQE30 (Qiagen). Recombinant proteins were produced using the QIAGEN protocol 7. Protein expression was induced for 5 hours by adding isopropyl-β-d-thiogalactopyranoside (IPTG) to a final concentration of 1 mM. Cells were harvested, and cell pellets were resuspended in lysis buffer (50 mM NaH_2_PO_4_, 300 mM NaCl, and 10 mM imidazole), followed by incubation with 1 mg/ml lysozyme for 30 min on ice and sonication. Cellular debris was removed by centrifugation, and the supernatant was incubated with Ni-nitrilotriacetic acid (NTA) agarose at 4°C for 3 h. beads were washed with 50 mM NaH_2_PO_4_, 300 mM NaCl, 60 mM imidazole, and 20% glycerol, and recombinant protein was eluted with 50 mM NaH_2_PO_4_, 300 mM NaCl, 250 mM imidazole, and 20% glycerol. The recombinant proteins were dialyzed against 20 mM HEPES, 200 mM NaCl, 17% glycerol, and stored at −80°C before use.

### Flow cytometry

Cells were analyzed by flow cytometry for the expression of CD38 (CD38-PE antibody, Santa Cruz 18,858, CD59 (PE-conjugated mouse anti-human CD59; BD Biosciences 555,764) and CD46 (PE-anti human CD46; R&D, FAB2005P). Cells were harvested from the suspension culture and incubated in a staining buffer (2% FBS in PBS) containing 0.5 μg/100 μl antibody for 30 min at room temperature in the dark, then washed once with staining buffer and resuspended in staining buffer for measurement on a FACSymphony A3 cell analyzer (BD Biosciences). The following antibodies were used: APC mouse anti-human CD46 (BD Biosciences), PE mouse anti-human CD59 (BD Biosciences), and PE anti-human CD38 (BioLegend).

### Sodium dodecyl sulfate-polyacrylamide gel electrophoresis

Samples were either boiled at 95°C for 5 min in a water bath to evaluate Ad35K++ monomers or not boiled to evaluate Ad35K++ homotrimers. Laemmli sample buffer and β-mercaptoethanol were added to all the samples. A total of 5 μg of protein sample was loaded per lane on 4–15% Mini-PROTEAN TGX gels. The gels were stained with Coomassie Blue.

### Complement-dependent cell killing assay (CDC)

A total of 150 μl of test cells (1.5 × 10^5^ cells/ml) was incubated with 5 μl PBS, Ad35K++, or rILYd4 at the concentrations indicated in the figure legends. Twelve hours later, 15 μg/ml daratumumab or isatuximab was added to the cells and incubated at room temperature for 30 min. Normal human serum was added to the final dilutions indicated in the figure legends. cells were then incubated at 37°C for another 3 h. The viable cells in each well were counted after trypan blue staining. For each data set shown in Figures 2, 3, 4, 5b, & 6, three independent experiments with three technical replicas were performed.

### Animal studies

All experiments involving animals were conducted in accordance with the institutional guidelines set forth by the University of Washington and were approved by the University Institutional Animal Care and Use Committee (IACUC protocol 3108–01). Mice were housed in specific-pathogen – free facilities. To establish the xenograft lymphoma model, 2 × 10^6^ MOLP8 cells in 200 μl of PBS was injected into the tail vein of immunodeficient severe combine immunodeficient (SCID)/beige (C.B-17/IcrHsd-scid-bg) mice. On day 8, 11 and 14, animals were intravenously injected with PBS, 50 µg Ad35K++ and rILYd4. Daratumumab or PBS were given intravenously 10 hours later. The percentage of human CD38^+^ cells in BM-MNCs was analyzed on day 27 after injection of MOLP8 cells.

### Statistical analyses

Statistical significance was calculated by two-tailed Student’s t-test. For comparisons of multiple groups, one-way and two-way analysis of variance (ANOVA) with Bonferroni post-testing for multiple comparisons were employed. Statistical analysis was performed using GraphPad Prism version 9.0.0 (GraphPad Software Inc., La Jolla, CA). *p* values: ** p < .05, ***p < .01, **** p < .001, n.s. not significant.

## Supplementary Material

Suppl_Figs_1_rev.docx

## Data Availability

All raw data will be made available by request.
