# Supplementary material for: CD46 and CD59 inhibitors enhance complement-dependent cytotoxicity of anti-CD38 monoclonal antibodies daratumumab and isatuximab in multiple myeloma and other B-cell malignancy cells
Source: Cancer Biol Ther. 2024 Feb 15;25(1):2314322. doi: 10.1080/15384047.2024.2314322 (PMC10877974; doi:10.1080/15384047.2024.2314322)
Supplement: Suppl_Figs_1_rev.docx [file KCBT_A_2314322_SM4804.docx]

# A B C


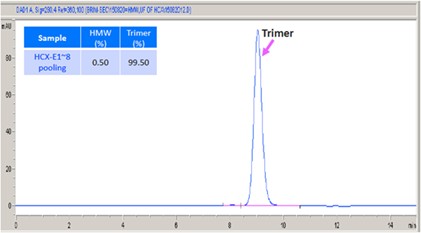

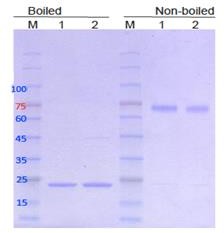

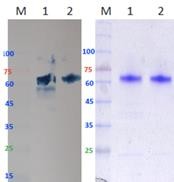


**Suppl. Fig.1. Characterization Ad35K++: A)** Size-exclusion HPLC of the final Ad35K++ preparation. The peak for the trimeric Ad35K++ fiber knob is labelled. **B)** 4-15% reduced SDS-Polyacrylamid gel electrophoresis. The mercaptoethanol-treated and boiled Ad35K++ dissociated into knob monomers with a MW of ~23 kDa. Without boiling, Ad35K++ runs as a trimer with a MW of ~68 kDa. M: Pre-stained markers. **C)** Western blot demonstrating Ad35K++ detection through binding to soluble CD46 and anti-CD46 antibodies. M: Pre-stained marker; 1: Ad35K++ interim reference standard; 2: Ad35K++ drug substance.

# A B

BJAB (1x105)


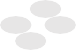

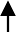


rILYd4 (2.5 μg/ml)

1 hours

30 min

rituxumab (15 μg/ml)


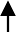


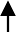


normal human

3 hours Cell viability (trypan blue exclusion)

(1) (2)


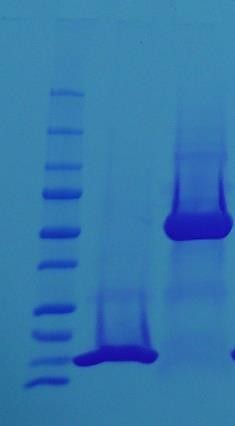


serum (NHS) (final 20%)

120

*

50kDa

100

80

% of viable cells

60

40

15kDa

20

0

Ctrl Rit/NHS rILYd4 rILYd4/Rit/NHS

**Suppl. Fig.2. Characterization of rILYd4. A)** SDS PAG analysis of recombinant rILYd4 and full-length rILY. Both proteins were produced from pQE30 plasmids in E.coli and purified via the N-terminal His-tag. The sequence of the 114 aa rILYd4 is MRGSHHHHHHGS- galtlnhdgafvarfyvyweelghdadgyetirsrswsgngynrgahysttlrfkgnvrnirvkvlgatglawepwrliyskndlplvpqrnistwgttlhpqfedkvvkdntd. The capital letters represent the His-tag. The theoretical molecular weight is 14.5kDa (lane 1). Lane 2 shows the full-length ILY(499 a.a.) with a theoretical molecular weight of 56.4kDa. **B)** Effect of rILYd4 on CDC triggered by rituximab (Rit) in human CD20+ lymphoma BJAB cells. * p<0.05 .

# A

120

****

***

ns

100

80

% of viable cells

60

120

100

**

*

ns

80

60

**B**

120

****

**

ns

100

80

% of viable cells

60

120

100

****

***

*

80

60

40 40

% of viable cells

40 40

20 20

% of viable cells

0 0

20 20

0 0

**Suppl. Fig.3 Titration of Ad35K++ and rILYd4.** Multiple myeloma MOLP8 cells were cultured with RPMI1640 supplemented with 10% heat-inactivated FBS. Cells were pre-incubated without and with increasing concentrations of Ad35K++ protein (A) or rILYd4 protein (B) for 14 hours. Daratumumab (Dara, 15 μg/ml) or isatuximab (Isa, 15 μg/ml) were added to cells and incubated at room temperature for 30 minutes. Then, normal human serum (NHS) was added, and cells were incubated at 37°C for another 3 hours. Viable cells in each well were counted after trypan blue staining. Each sample was performed with biological duplicates and counted 4 times (8 times total). Based on this study, we decided to perform all tests with 2.5μg/ml Ad35K++ or rILYd4.
